# Proteomic Analysis of *Hylocereus polyrhizus* Reveals Metabolic Pathway Changes

**DOI:** 10.3390/ijms17101606

**Published:** 2016-09-28

**Authors:** Qingzhu Hua, Qianjun Zhou, Susheng Gan, Jingyu Wu, Canbin Chen, Jiaqiang Li, Yaoxiong Ye, Jietang Zhao, Guibing Hu, Yonghua Qin

**Affiliations:** 1 State Key Laboratory for Conservation and Utilization of Subtropical Agro-Bioresources/Key Laboratory of Biology and Genetic Improvement of Horticultural Crops-South China, Ministry of Agriculture, College of Horticulture, South China Agricultural University, Guangzhou 510642, China; huaqingzhu1010@gmail.com (Q.H.); wujingyuscau@163.com (J.W.); nnchencanbin@163.com (C.C.); zhaojietang@gmail.com (J.Z.); 2 General Station of the Administration of Seeds Guangdong Province, Guangzhou 510500, China; zzzzglk@163.com; 3 Plant Biology Section, School of Integrative Plant Science, Cornell University, Ithaca, NY 14853, USA; sg288@cornell.edu; 4 Dongguan Institute of Forest Science, Dongguan 523106, China; jqli618@163.com (J.L.); yxyeforest@163.com (Y.Y.)

**Keywords:** *Hylocereus polyrhizus*, isobaric tags for relative and absolute quantitation (iTRAQ), transcriptome, betalain biosynthesis

## Abstract

Red dragon fruit or red pitaya (*Hylocereus polyrhizus*) is the only edible fruit that contains betalains. The color of betalains ranges from red and violet to yellow in plants. Betalains may also serve as an important component of health-promoting and disease-preventing functional food. Currently, the biosynthetic and regulatory pathways for betalain production remain to be fully deciphered. In this study, isobaric tags for relative and absolute quantitation (iTRAQ)-based proteomic analyses were used to reveal the molecular mechanism of betalain biosynthesis in *H. polyrhizus* fruits at white and red pulp stages, respectively. A total of 1946 proteins were identified as the differentially expressed between the two samples, and 936 of them were significantly highly expressed at the red pulp stage of *H. polyrhizus*. RNA-seq and iTRAQ analyses showed that some transcripts and proteins were positively correlated; they belonged to “phenylpropanoid biosynthesis”, “tyrosine metabolism”, “flavonoid biosynthesis”, “ascorbate and aldarate metabolism”, “betalains biosynthesis” and “anthocyanin biosynthesis”. In betalains biosynthesis pathway, several proteins/enzymes such as polyphenol oxidase, CYP76AD3 and 4,5-dihydroxy-phenylalanine (DOPA) dioxygenase extradiol-like protein were identified. The present study provides a new insight into the molecular mechanism of the betalain biosynthesis at the posttranscriptional level.

## 1. Introduction

Betalains are red and yellow pigments in plants of Caryophyllales only. It is interesting that betalains and anthocyanins are naturally mutually exclusive in an individual plant. Betalain pigments have potential benefits in promoting health and preventing diseases of human being by serving as potent antioxidant and possessing anti-inflammatory and chemo-preventive activities in vitro and in vivo [1,2,3,4,5,6]. Betalains also contribute to the early-phase insulin response [7] and play a role in dye-sensitized solar cells (DSSCs) [8,9,10]. The biosynthesis of betalains has become one of the hot research topics due to its high nutritional properties and bioactivities.

Betalain biosynthesis is influenced by various factors such as light, temperature, nutrition, plant growth regulators and enzymatic activities. Light is important for betalain biosynthesis in *Amaranthus*, *Caudatus*, *Portulaca* and *Suaeda* [11,12,13,14,15,16]. In the presence of light, cytokinin, ethylene and methyl jasmonate (MeJA) can enhance betacyanin accumulation in species of *Amaranthus*, *Beta vulgaris*, *Portulaca*, *Suaeda* and *Mesembryanthemum* [14,17,18,19,20,21,22]. Betalains can also be accumulated in adverse circumstances such as salinity and drought stress [23,24,25], which are attributed to defense against biotic and abiotic stresses [26].

The key enzymes involved in the betalain biosynthetic pathways have been known. There are five pathways derived from tyrosine and four pathways derived from tyramine [27,28]. Except for enzymatic reactions, the conjugation reactions of pigment formation are assumed to occur spontaneously [28,29,30]. Enzymes involved in betalain biosynthesis in higher plants are usually divided into three classes, namely tyrosinase (TYR), 4,5-dihydroxy-phenylalanine (DOPA)-dioxygenase (DOD), and glucosyltransferases (GTs). TYR plays a key role in hydroxylation and oxidation to form L-DOPA, *cyclo*-DOPA, tyr-betaxanthin and dopaxanthin that are the pivotal precursors of betalain biosynthesis [12,29,31,32,33]. TYR sequences have been obtained from *Phytolacca* *americana* [34], *Spinacia oleracea* [35] and *Suaeda salsa* [36]. The first PPO promoter was identified in *B. vulgaris* subsp. *cicla* [37]. DOD is a key enzyme catalyzing the cleavage of L-DOPA needed for the formation of betalamic acid. Compared to other enzymes, DOD has been fully studied in betalain-producing plant species such as *Amaranthus hypochondriacus* [38], *Portulaca grandiflora* [39,40], *Suaeda salsa* [41,42], *B. vulgaris* [43], *Opuntia ficus-indica* [44], *Parakeelya mirabilis* [45], *Mirabilis jalapa* and *Bougainvillea glabra* [46]. CYP76AD, a novel cytochrome P450 enzyme, catalyzes oxidation of L-DOPA for the formation of cyclo-DOPA in *B. vulgaris* [47], *A. hypochondriacus* [38] and *M. jalapa* [48]. In addition, *CYP76AD* and *DOD* have also been considered as key genes involved in betalain biosynthesis based on phylogenetic analysis [49,50]. GTs are the key enzyme in betalain biosynthetic pathway to make the pigments stable and diversified. GT sequences have been obtained from *B. vulgaris* [51], *O. ficus-indica* [44], *P. americana* [52] and *A. hypochondriacus* [38], and there have been no further major advances in GTs since they were identified as B5GT (betanidin-5-O-GT occurred on betanidin 5-O location), B6GT (betanidin-6-O-GT occurred on betanidin 6-O location) and CDOPA5GT (cyclo-DOPA 5-O-GT occurred on cyclo-DOPA) [53,54,55]. Recently, the first regulatory gene (a R2R3 *MYB*) associated with regulation of the betalain biosynthetic pathway was identified in this step [56,57].

Dragon fruit or pitaya is one of the tropical fruits belonging to *Hylocereus* in the Cactaceae family. Pitaya is cultivated in a wide ecological range due to its tolerance to such environmental cues as drought, heat and poor soil [58,59]. Pitaya is characterized by its excellent nutritional, commercial and medical value [60,61]. It has become one of the newly cultivated fruits in Thailand, Philippines, Vietnam, Malaysia and China. Red pulp pitaya (*H. polyrhizus*) is the only fruit that contains betalains [62,63,64]. Great progress has been made in betalain purification, identification and biosynthesis-related compound analyses [65,66,67]. However, the key enzymes or genes involved in the betalain biosynthesis pathways in pitaya remain elusive. Recently, we obtained nine putative betalain biosynthesis-related genes by analyzing the transcriptomic data of *H. polyrhizus* [68]. Here we used isobaric tags for relative and absolute quantitation (iTRAQ) to identify key proteins/enzymes that are differentially expressed in pitaya fruit at white vs. red pulp stages in *H.* *polyrhizus*, with a focus on those proteins potentially involved in the betalain biosynthesis in pulp coloration of this fruit.

## 2. Results and Discussion

### 2.1. Overview of the Proteomics Analysis of the Pitaya Fruit Development

iTRAQ is a recently developed technique in quantitative proteomics that very accurately measures large-fold changes in protein expression within broad, dynamic ranges of protein abundance [69]. Here we used this technique to perform comparative proteomic analyses of the key enzymes involved in betalain biosynthetic pathways in pitaya. For the proteomic analysis, two proteomic libraries were constructed using the pitaya (*H.* *polyrhizus*) fruits at two developmental stages (white and red pulps). Total proteins and changes in the protein profile upon coloring process were explored using the iTRAQ technique. A total of 89,583 peptides were identified that belong to 33,548 peptide species. In total, 6725 proteins were identified and quantified under the condition of 1% false-discovery rate (FDR) and only those unique peptides were used for quantitative comparison. A threshold of ≥1.5 for fold changes (FC) was set to filter the comparative data sets, resulting in the identification of 1946 proteins as the differentially expressed between the two samples. Among the differentially expressed proteins, 936 were significantly up-expressed in the fruits at the red pulp stage compared with those at the white stage. iTRAQ has the advantages of high coverage, accuracy and sensitivity, and our proteomic analyses of *H.* *polyrhizus* fruits using the iTRAQ technique could provide useful information for understanding the pitaya fruit development in general and the betalain biosynthesis pathway in particular as discussed below (Table 1 and Table 2).

### 2.2. Functional Classification of Differentially Expressed Proteins during Pitaya Fruit Coloring

**Figure 1:**
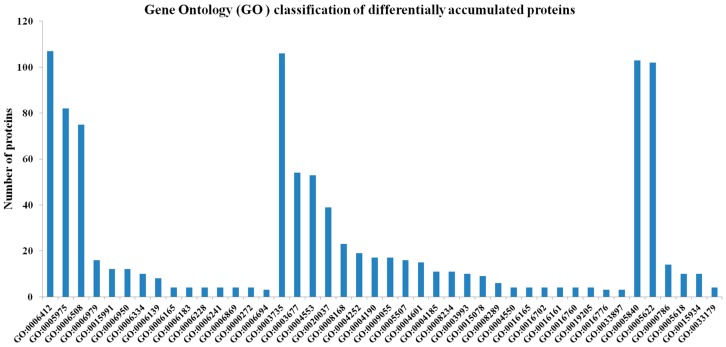
Gene Ontology (GO) classification of differentially accumulated proteins during pitaya fruit ripening. GO:0006412//translation, GO:0005975//carbohydrate metabolic process, GO:0006508//proteolysis, GO:0006979//response to oxidative stress, GO:0015991//ATP hydrolysis coupled proton transport, GO:0006950//response to stress, GO:0006334//nucleosome assembly, GO:0006139//nucleobase-containing compound metabolic process, GO:0006165//nucleoside diphosphate phosphorylation, GO:0006183//GTP, biosynthetic process, GO:0006228//UTP biosynthetic process, GO:0006241//CTP biosynthetic process, GO:0006869//lipid transport, GO:0000272//polysaccharide catabolic process, GO:0006694//steroid biosynthetic process, GO:0003735//structural constituent of ribosome, GO:0003677//DNA binding, GO:0004553//hydrolase activity, hydrolyzing *O*-glycosyl compounds, GO:0020037//heme binding, GO:0008168//methyltransferase activity, GO:0004252//serine-type endopeptidase activity, GO:0004190//aspartic-type endopeptidase activity, GO:0009055//electron carrier activity, GO:0005507//copper ion binding, GO:0004601//peroxidase activity, GO:0004185//serine-type carboxypeptidase activity, GO:0008234//cysteine-type peptidase activity, GO:0003993//acid phosphatase activity, GO:0015078//hydrogen ion transmembrane transporter activity, GO:0008289//lipid binding, GO:0004550//nucleoside diphosphate kinase activity, GO:0016165//linoleate 13S-lipoxygenase activity, GO:0016702//oxidoreductase activity, acting on single donors with incorporation of molecular oxygen, incorporation of two atoms of oxygen, GO:0016161//beta-amylase activity, GO:0016760//cellulose synthase (UDP-forming) activity, GO:0019205//nucleobase-containing compound kinase activity, GO:0016776//phosphotransferase activity, phosphate group as acceptor, GO:0033897//ribonuclease T2 activity, GO:0005840//ribosome, GO:0005622//intracellular, GO:0000786//nucleosome, GO:0005618//cell wall, GO:0015934//large ribosomal subunit, GO:0033179//proton-transporting V-type ATPase, V0 domain.

**Figure 2:**
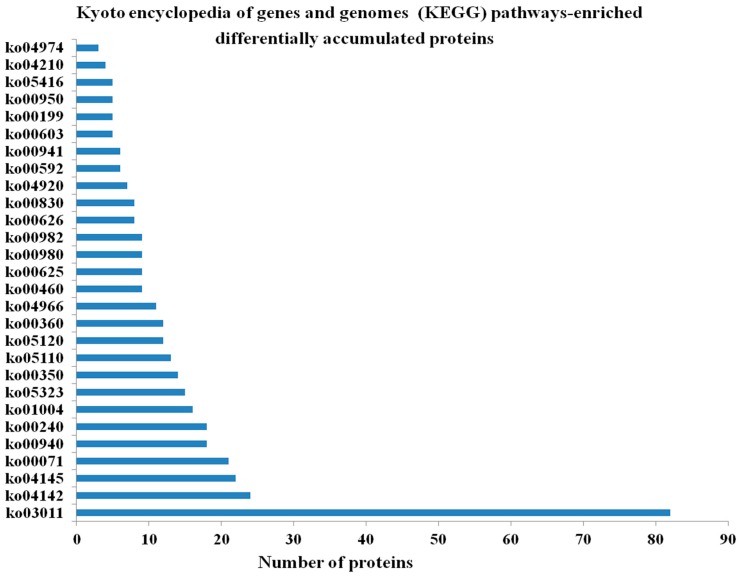
Kyoto encyclopedia of genes and genomes(KEGG) pathways-enriched differentially accumulated proteins during pitaya fruit ripening. ko03011 Ribosome, ko04142 Lysosome, ko04145 Phagosome, ko00071 Fatty acid metabolism, ko00940 Phenylpropanoid biosynthesis, ko00240 Pyrimidine metabolism, ko01004 Lipid biosynthesis proteins, ko05323 Rheumatoid arthritis, ko00350 Tyrosine metabolism, ko05110 Vibrio cholerae infection, ko05120 Epithelial cell signaling in Helicobacter pylori infection, ko00360 Phenylalanine metabolism, ko04966 Collecting duct acid secretion, ko00460 Cyanoamino acid metabolism, ko00625 Chloroalkane and chloroalkene degradation, ko00980 Metabolism of xenobiotics by cytochrome P450, ko00982 Drug metabolism—cytochrome P450, ko00626 Naphthalene degradation, ko00830 Retinol metabolism, ko04920 Adipocytokine signaling pathway, ko00592 alpha-Linolenic acid metabolism, ko00941 Flavonoid biosynthesis, ko00603 Glycosphingolipid biosynthesis - globo series, ko00199 Cytochrome P450, ko00950 Isoquinoline alkaloid biosynthesis, ko05416 Viral myocarditis, ko04210 Apoptosis, ko04974 Protein digestion and absorption.

The proteomes representing the protein profiles of the pitaya fruits at the white or red pulp stages were comparatively analyzed based on the assigned functions of the proteins in the public protein databases. Five hundred and seven (507) differentially accumulated proteins (*p*-value < 0.05, fold change (FC) > 1.5) in the red pulp fruits were annotated and classified into “biological process (BP)”, “molecular function (MF)”, and “cellular component (CC)” categories as well as their sub-categories. Based on the numbers of unique proteins identified in each of the functional categories, the two largest categories for each functional group were as follows: “translation” and “carbohydrate metabolic processes” for BP; “structural constituent of ribosome” and “DNA binding” for MF; and “ribosome” and “intracellular” for CC (Figure 1). Some of these proteins could be mapped to the following pathways: “phenylpropanoid biosynthesis”, “phenylalanine metabolism”, “tyrosine metabolism” and “flavonoid biosynthesis” (Figure 1). These pathways might have impacts on the formation of betalain. Two hundred eighteen (218) proteins were assigned to 23 kyoto encyclopedia of genes and genomes (KEGG) pathways. The most frequently detected or abundant proteins, representing 37.6% of all the protein identified, belonged to the pathway of “ribosome” (Figure 2). Further work is needed to establish the metabolic profiles in red pitaya at a series of developmental stages.

### 2.3. Integrated Analyses of Transcriptomic and Proteomic Datasets on the Pitaya Ripening

In our previous study, the high efficient RNA sequencing (RNA-Seq) technology was used to identify key genes related to betalain biosynthesis during pulp coloration of *H. polyrhizus*. A total of about 12 Gb raw RNA-Seq data were generated and de novo assembled into 122,677 transcripts, of which 122,668 were annotated [68]. Differentially expressed transcripts and proteins between the two stages were identified by comparing the RNA-seq and iTRAQ datasets. Although most of the differentially expressed were consistent at both the transcript and protein levels (see below), discrepancy was detected between the transcript and protein data, and such genes/proteins fell into the following two groups. The first group is those differentially expressed at the transcript level but not at the protein level (*p*-value < 0.05, FC (pro) < 1.3, FC (RNA) > 2). They functionally belonged to “oxidation-reduction process” (BP); “oxidoreductase activity, acting on paired donors, with incorporation or reduction of molecular oxygen” (MF); and “protein complex” (CC) (Table S1). These differentially expressed were further analyzed using the KEGG database, and fell into 12 pathways, with five differentially-accumulated proteins associated with the “arginine metabolism” (Table S2). The second group is those differentially expressed at the protein level but not at the transcript level (*p*-value < 0.05, FC (pro) > 1.5, FC (RNA) < 2). However, no protein matching with corresponding transcript were detected. Analyzing the Gene Ontology (GO) categories and KEGG pathways of the differentially expressed with a negative correlation between the transcript and protein levels allowed us to map 22 differentially expressed genes/proteins (Tables S3 and S4). The fact that the abundance of transcripts differed from that of respective proteins strongly suggested that there were posttranscriptional regulation involved in the metabolisms and other cellular processes associated with the pitaya ripening. In fact, similar findings have previously been found in many other biological processes such as in human [70], yeast [71] and plants [72]. Such findings became possible only when the transcriptomic and proteomic datasets were integrated.

**Figure 3:**
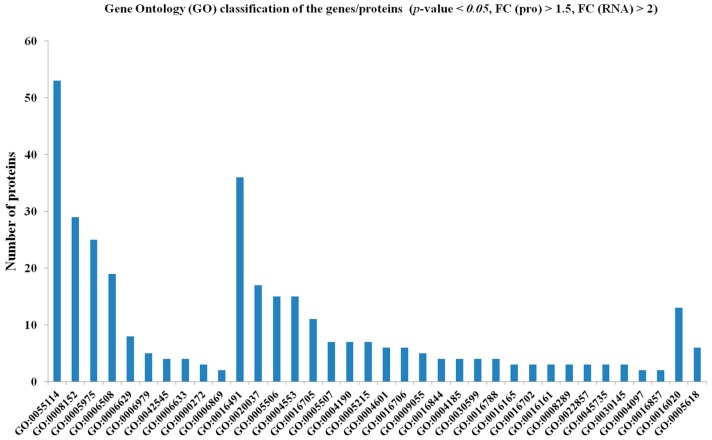
Gene Ontology (GO) classification of the genes/proteins whose transcript and protein levels were positively correlated (*p*-value < 0.05, FC (pro) > 1.5, FC (RNA) > 2) during the pitaya fruit ripening. GO:0055114//oxidation-reduction process, GO:0008152//metabolic process, GO:0005975//carbohydrate metabolic process, GO:0006508//proteolysis, GO:0006629//lipid metabolic process, GO:0006979//response to oxidative stress, GO:0042545//cell wall modification, GO:0006633//fatty acid biosynthetic process, GO:0000272//polysaccharide catabolic process, GO:0006869//lipid transport, GO:0016491//oxidoreductase activity, GO:0020037//heme binding, GO:0005506//iron ion binding, GO:0004553//hydrolase activity, hydrolyzing *O*-glycosyl compounds, GO:0016705//oxidoreductase activity, acting on paired donors, with incorporation or reduction of molecular oxygen, GO:0005507//copper ion binding, GO:0004190//aspartic-type endopeptidase activity, GO:0005215//transporter activity, GO:0004601//peroxidase activity, GO:0016706//oxidoreductase activity, acting on paired donors, with incorporation or reduction of molecular oxygen, 2-oxoglutarate as one donor, and incorporation of one atom each of oxygen into both donors, GO:0009055//electron carrier activity, GO:0016844//strictosidine synthase activity, GO:0004185//serine-type carboxypeptidase activity, GO:0030599//pectinesterase activity, GO:0016788//hydrolase activity, acting on ester bonds, GO:0016165//linoleate 13S-lipoxygenase activity, GO:0016702//oxidoreductase activity, acting on single donors with incorporation of molecular oxygen, incorporation of two atoms of oxygen, GO:0016161//beta-amylase activity, GO:0008289//lipid binding, GO:0022857//transmembrane transporter activity, GO:0045735//nutrient reservoir activity, GO:0030145//manganese ion binding, GO:0004097//catechol oxidase activity, GO:0016857//racemase and epimerase activity, acting on carbohydrates and derivatives, GO:0016020//membrane, GO:0005618//cell wall.

Despite the discrepancy discussed above, most of the genes/proteins had a positive correlation (*p*-value < 0.05, FC (pro) > 1.5, FC (RNA) > 2), i.e., differentially expressed at both transcript and protein levels. These genes/proteins were mapped to such categories as “oxidation-reduction process” (BP), “oxidoreductase activity” (MF) and “membrane” (CC) (Figure 3). There were totally 15 pathways were obtained, including “starch and sucrose metabolism”, “phenylpropanoid biosynthesis”, “lipid biosynthesis proteins”, “ascorbate and aldarate metabolism”, “tyrosine metabolism” and “flavonoid biosynthesis” (Table 1).

Among the positively correlated genes/proteins, three were up-regulated in “starch and sucrose metabolism”. It was reported that soluble solids concentration (SSC) was closely related to the synthesis of betacyanin [73]. Five up-regulated genes/proteins at both the transcriptional and posttranscriptional levels were in “phenylpropanoid biosynthesis”. Two up-regulated and two down-regulated genes/proteins belonged to the “tyrosine metabolism” pathway. Betalains were derived from tyrosine [28], and the “phenylpropanoid biosynthesis” had been considered to be the upstream pathway of the tyrosine pathway. Two transcripts and proteins involved in “flavonoid biosynthesis” were significantly up-regulated and down-regulated, respectively. Four up-regulated transcripts and proteins were enriched in “ascorbate and aldarate metabolism” (Table 2), which played a role in the biosynthesis of betalains [28] (Table 2). These proteins may be associated with the betalain biosynthesis in *H. polyrhizus*.

Naturally, betalains and anthocyanin could not co-exist simultaneously in one plant. However, they can exist together by genetic engineering strategy [30,31]. Chalcone isomerase (CHI) and chalcone synthase (CHS) were the upstream enzymes of the anthocyanin biosynthesis pathway. In this study, one protein annotated as CHI and another as CHS were identified. CHI and CHS had higher expressed levels in the pitaya fruit at the red stage than at the white stage. This result was consistent with the conjecture that betalain-producing plants could not produce anthocyanins due to lower levels of dihydroflavonol reductase (DFR), anthocyanidin synthase (ANS) and leuco anthocyanidin reductase (LAR) [74]. More interestingly, five proteins, namely comp37375_c0_seq2_4 and comp37375_c0_seq1_4 (polyphenol oxidase), comp37692_c0_ seq1_7 (4, 5-DOPA dioxygenase extradiol-like), comp16058_c0_seq1_4 (CYP76AD3), and comp26435_c0_seq1_2 (Aromatic-l-amino-acid decarboxylase), were identified as some of the key enzymes in the betalain biosynthesis pathway (Table 2). Not surprisingly, the transcriptional levels of these proteins were increasing with the progression of the betalain formation in the pitaya fruit as revealed in our previous study [68].

These results suggested that these pathways could be involved in the biosynthesis of pigments, and it was possible that polyphenol oxidase, CYP76AD3 and 4,5-DOPA dioxygenase extradiol-like protein were responsible for betalain biosynthesis in the pitaya fruit. These results were consistent with our previous findings from transcriptomic analysis that these enzymes might be involved in betalain biosynthesis in *H.* *polyrhizus* [68].

## 3. Materials and Methods

### 3.1. Plant Material

“7-1” (*H. polyrhizus*), a superior selection with red flesh color and excellent quality, were used as materials. Plants were cultivated in Dalingshan Forest Park (East longitude: 113°42′22″–113°48′12″, Northern latitudes: 22°50′00″–22°53′32″), Dongguan City, Guangdong Province, China. Two libraries from white and red pulp stages were constructed for iTRAQ analyses on the 21st and 32nd days after artificial pollination in July and August 2014 (Figure S1). Every library consisted of equal amounts of protein from three fruit at each fruit developmental stage.

### 3.2. Samples Preparation, Protein Extraction and Detection

Upon harvested, the samples described above were immediately frozen in liquid nitrogen and stored at −80 °C prior to protein extraction. Trichloroacetic acid (TCA)-acetone method was used for protein extraction. Briefly, pulps without seeds were ground to fine powder in liquid nitrogen. Cold TCA-acetone (ratio of material to liquid is 1:4) was added to the cold 15 mL tube and vortexed for 30 s. Total proteins were precipitated overnight at −20 °C. Precipitation was collected by spinning 12,000 rpm for 15 min at 4 °C and subsequently washed with cold acetone and 90% acetone, respectively. The precipitation was freeze-dried by vacuum and dissolved in lysis buffer [8 M ureophil, 0.5% SDS (sodium dodecyl sulfate), PMSF (phenylmethanesulfonyl fluoride), PI (protease inhibitor), 40 mM TEAB (Triethylamine borane)]. Proteins in the supernatant was pelleted by centrifuging 12,000 rpm for 15 min and extracted with 8 M ureophil extraction buffer (including 0.1% SDS, PI, PMSF). The concentration and quality of the protein were assayed by bicinchoninic acid (BCA) method and SDS-PAGE gel electrophoresis. At least 400 μg proteins (>2.0 μg/μL) were used for further experiment.

### 3.3. iTRAQ Labeling

Above extracted protein samples were prepared using the iTRAQ^®^ Reagents 8plexMulti-plex kit (AB Sciex, Boston, MA, USA). Enriched phosphopeptides were labeled with isobaric tags for relative and absolute quantification reagents (AB Sciex). iTRAQ 113 and 115 were used to label white and red samples, respectively. The labeled samples were combined equably and graded with RP C18 chromatographic column.

### 3.4. Strong Cation Exchange Chromatography (SCX)

The mixed and labeled samples were fractionated by SCX fractionation using HPLC (high performance liquid chromatography). The mobile phases consisted of buffer A (2% acetonitrile and 98% H_2_O (pH 10)) and B (90% acetonitrile and 10% H_2_O (pH 10)). The labeled samples were concentrated by vacuum and dissolved in 100 μL buffer A (pH 10). After the mixture was centrifuged, the supernatant was loaded onto a reverse phase (RP) C18 precolumn (LC Packings) (Agilent Technologies, Palo Alto, CA, USA). Separation was performed using a linear gradient at a flow rate of 1 mL/min. The gradient of elution is shown in Table S5. The liquid effluent was collected at a speed of 1.5 mL/min. Multiple components were obtained by merging samples according to the chromatogram map.

### 3.5. LC-MS/MS Analysis

The liquid chromatography–mass spectrometry (LC-MS)/MS method was developed for the separation and analysis of samples. The mass spectrometer used for detection was Q Exactive mass spectrometer (Thermo Scientific, Waltham, MA, USA). The HPLC was NCS3500 system. The mobile phases consisted of buffer A (99.9% H_2_O and 0.1% formic acid) and B (99.9% acetonitrile and 0.1% formic acid). The flow rate was 300 nL/min. The gradient of elution is as shown in Table S6.

Full MS scans range was 350–1600 *m*/*z*. The runtime was 75 min and the resolution was 70,000. The precursor ions were selected for the MS/MS scans using higher energy collision-induced dissociation (HCD) for each precursor ion. Then MS2 (secondary mass spectrum) sequences were determined. The dynamic exclusion option was implemented with a repeat count of 1 and exclusion duration of 15 s. The values of automated gain control (AGC) were set to 1 × 10^6^ and 2 × 10^5^ for full MS and MS2, respectively.

### 3.6. Proteomic Data Analysis

For peptide data analysis, raw mass data were processed and searched against the protein databases downloaded from the public databases using Proteome Discoverer software (Thermo Scientific, Waltham, MA, USA). Searches were performed using the following criteria: the precursor mass tolerance was set to 20 ppm, and fragment ion mass tolerance was set to 0.02 Da for HCD in addition to the general settings. The search parameters allowed two missed cleavage for tyrpsin. The maximum delta Cn was considered as 0.05, while 10 was thre maximum number of peptides reported. The score threshold for peptide identification was set at 0.01 false-discovery rate (FDR) in the iTRAQ experiment. The list of proteins obtained from the iTRAQ data was exported to Supplementary File S1, which contains such protein-specific information as accession numbers, percent coverage, protein scores, number of peptides matching individual proteins, etc. Proteins with a fold-change cutoff ≥1.5 between the two stages were identified as differentially expressed.

## 4. Conclusions

Betalains play a role in appearance quality and nutritional value of red pitaya, and they are also involved in stress-resistance. Study of the pitaya pulp pigments proteome is important to understand the correlative pathways contributed to the betalain biosynthesis. In this study, proteomic changes in *H.* *polyrhizus* were first investigated using iTRAQ. In total, 1946 differentially expressed proteins were identified to characterize the proteome of red pitaya. Based on integrated analyses of transcriptomic and proteomic datasets, several transcripts and proteins owning positive correlation were gathered in “phenylpropanoid biosynthesis”, “tyrosine metabolism”, “flavonoid biosynthesis”, “ascorbate and aldarate metabolism”, “betalains biosynthesis” and “anthocyanin biosynthesis”. These pathways are related to the metabolites of betalain biosynthesis. Five proteins, which were annotated to polyphenol oxidase, CYP76AD3, and 4,5-DOPA dioxygenase extradiol-like, were found to be differentially expressed in the betalain biosynthesis pathway. The present study provides the first proteomic analysis of the red pitaya pulps by iTRAQ and could offer new insights into the molecular mechanism of the betalain biosynthesis at the posttranscriptional level.

## Figures and Tables

**Table 1:** Kyoto encyclopedia of genes and genomes (KEGG) pathway enrichment of the genes/proteins with a positive correlation (*p*-value < 0.05, FC (pro) > 1.5, FC (RNA) > 2) between the levels of transcripts and proteins.

KEGG Pathway	No. All ^a^	No. Up ^b^	No. Down ^c^
Starch and sucrose metabolism	6	3	3
Phenylpropanoid biosynthesis	5	5	0
Lipid biosynthesis proteins	5	3	2
Fatty acid metabolism	5	5	0
Cysteine methionine metabolism	5	4	1
Amino sugar and nucleotide sugar metabolism	5	1	4
Ascorbate and aldarate metabolism	4	4	0
Tyrosine metabolism	4	2	2
Cyanoamino metabolism	3	0	3
Chloroalkane and chloroalkene degradation	3	3	0
Glycerolipid metabolism	3	3	0
Valine leucine isoleucine biosynthesis	3	0	3
Isoquinoline alkaloid biosynthesis	2	0	2
Flavonoid biosynthesis	2	1	1
Naphthalene degradation	2	2	0

^a^, the total number of differentially expressed on genes and proteins; ^b^, the number of significantly up-regulated expression in the pitaya fruit at the red pulp stage; ^c^, the number of significantly down-regulated expression in the pitaya fruit at the red pulp stage. No., number; FC, fold changes.

**Table 2:** Proteins enriched in several KEGG pathways with a positive correlation (*p*-value < 0.05, FC (pro) > 1.5, FC (RNA) > 2) between the levels of transcripts and proteins.

Accession No.	Annotation	Ratio (32rd day vs. 21st day)
**Starch and sucrose metabolism**		
comp28763_c0_seq2_8	K00688 starch phosphorylase (EC:2.4.1.1)	2.218
comp31213_c0_seq1_12	K05350 β-glucosidase (EC:3.2.1.21)	0.444
comp34816_c0_seq1_6	K00695 sucrose synthase (EC:2.4.1.13)	2.163
comp34279_c0_seq1_6	K05350 β-glucosidase (EC:3.2.1.21)	0.435
comp35284_c0_seq1_11	K01051 pectinesterase (EC:3.1.1.11)	2.531
comp37752_c0_seq1_2	K00695 sucrose synthase (EC:2.4.1.13)	0.261
**Phenylpropanoid biosynthesis**		
comp21956_c1_seq1_1	K12355 coniferyl-aldehyde dehydrogenase (EC:1.2.1.68)	0.437
comp21956_c2_seq1_2	K12355 coniferyl-aldehyde dehydrogenase (EC:1.2.1.68)	0.465
comp31213_c0_seq1_12	K05350 β-glucosidase (EC:3.2.1.21)	0.444
comp32862_c0_seq1_3	K09754 p-coumarate 3-hydroxylase (EC:1.14.13.-)	0.651
comp34279_c0_seq1_6	K05350 β-glucosidase (EC:3.2.1.21)	0.435
**Ascorbate and aldarate metabolism**		
comp21409_c0_seq1_2	K00434 l-ascorbate peroxidase (EC:1.11.1.11)	1.961
comp27881_c0_seq1_8	K00128aldehyde dehydrogenase (NAD+) (EC:1.2.1.3)	2.211
comp28683_c0_seq1_3	K00434 l-ascorbate peroxidase (EC:1.11.1.11)	1.618
comp31481_c0_seq1_3	K08232 monodehydroascorbate reductase (NADH) (EC:1.6.5.4)	1.696
**Tyrosine metabolism**		
comp23153_c0_seq1_3	K00001 alcohol dehydrogenase (EC:1.1.1.1)	3.612
comp25940_c0_seq3_7	K00813 aspartate aminotransferase (EC:2.6.1.1)	0.589
comp26435_c0_seq1_2	K01592 tyrosine decarboxylase (EC:4.1.1.25)	0.637
comp27557_c0_seq1_3	K00001 alcohol dehydrogenase (EC:1.1.1.1)	7.142
**Flavonoid biosynthesis**		
comp29018_c0_seq1_2	K01859 chalcone isomerase (EC:5.5.1.6)	2.205
comp32862_c0_seq1_3	K09754 p-coumarate 3-hydroxylase (EC:1.14.13.-)	0.651
**Anthocyanin biosynthesis**		
comp29018_c0_seq1_2	K01859 chalcone isomerase (EC:5.5.1.6)	2.205
comp24963_c0_seq1_6	K00660 chalcone synthase (EC:2.3.1.74)	1.556
**Betalains biosynthesis**		
comp16058_c0_seq1_4	CYP76AD3 (*Mirabilis jalapa*)	2.373
comp37692_c0_seq1_7	4,5-DOPA dioxygenase extradiol	3.035
comp37375_c0_seq2_4	Polyphenol oxidase	0.279
comp37375_c0_seq1_4	polyphenol oxidase	0.262
comp25579_c0_seq2_3	multicopper oxidase, putative	0.419
comp26435_c0_seq1_2	Aromatic-l-amino-acid decarboxylase	0.637

## Supplementary Materials

Supplementary materials can be found at www.mdpi.com/1422-0067/17/10/1606/s1.

## References

[1] Clifford T., Howatson G., West D.J., Stevenson E.J. (2015). The potential benefits of red beetroot supplementation in health and disease. Nutrients.

[2] Nowacki L.T., Vigneron P., Rotellini L., Cazzola H.L.N., Merlier F., Prost E., Ralanairina R., Gadonna J., Rossi C., Vayssade M. (2015). Betanin-enriched red beetroot (*Beta vulgaris* L.) extract induces apoptosis and autophagic cell death in MCF-7 cells. Phytother. Res..

[3] Martinez R.M., Longhi-Balbinot D.T., Zarpelon A.C., Staurengo-Ferrari L., Baracat M.M., Georgetti S.R., Sassonia R.C., Verri W.A., Casagrande R. (2015). Anti-inflammatory activity of betalain-rich dye of *Beta vulgaris*: Effect on edema, leukocyte recruitment, superoxide anion and cytokine production. Arch. Pharm. Res..

[4] Naselli F., Tesoriere L., Caradonna F., Bellavia D., Attanzio A., Gentile C., Livrea M.A. (2014). Anti-proliferative and pro-apoptotic activity of whole extract and isolated indicaxanthin from *Opuntia ficus-indica* associated with re-activation of the onco-suppressor p16(INK4a) gene in human colorectal carcinoma (Caco-2) cells. Biochem. Biophys. Res. Commun..

[5] Allegra M., Ianaro A., Tersigni M., Panza E., Tesoriere L., Livrea M.A. (2014). Indicaxanthin from cactus pear fruit exerts anti-inflammatory effects in carrageenin-induced rat pleurisy. J. Nutr..

[6] Allegra M., Tesoriere L., Livrea M.A. (2007). Betanin inhibits the myeloperoxidase/nitrite-induced oxidation of human low-density lipoproteins. Free Radic. Res..

[7] Wootton-Beard P.C., Brandt K., Fell D., Warner S., Ryan L. (2014). Effects of a beetroot juice with high neobetanin content on the early-phase insulin response in healthy volunteers. J. Nutr. Sci..

[8] Sengupta D., Mondal B., Mukherjee K. (2015). Visible light absorption and photo-sensitizing properties of spinach leaves and beetroot extracted natural dyes. Spectrochim. Acta A.

[9] Hernandez-Martinez A.R., Estevez M., Vargas S., Quintanilla F., Rodriguez R. (2011). New dye-sensitized solar cells obtained from extracted bracts of *Bougainvillea glabra* and *spectabilis* betalain pigments by different purification processes. Int. J. Mol. Sci..

[10] Calogero G., Bartolotta A., di Marco G., di Carlo A., Bonaccorso F. (2015). Vegetable-based dye-sensitized solar cells. Chem. Soc. Rev..

[11] Boo H., Shin K., Heo J., Jeong J., Paek K. Betalain synthesis by hairy root of red beet cultured in vitro under different light quality. Proceedings of the 4th International ISHS Symposium on Artificial Lighting.

[12] Wang C.Q., Song H., Gong X.Z., Hu Q.G., Liu F., Wang B.S. (2007). Correlation of tyrosinase activity and betacyanin biosynthesis induced by dark in C3 halophyte *Suaeda salsa* seedlings. Plant Sci..

[13] Kishima Y., Nozaki K., Akashi R., Adachi T. (1991). Light-inducible pigmentation in *Portulaca* callus; selection of a high betalain producing cell line. Plant Cell Rep..

[14] Kishima Y., Shimaya A., Adachi T. (1995). Evidence that blue light induces betalain pigmentation in *Portulaca* callus. Plant Cell Tissue Org..

[15] Kochhar V.K., Kochhar S., Mohr H. (1981). An analysis of the action of light on betalain synthesis in the seedling of *Amaranthus caudatus*, var. viridis. Planta.

[16] Khandaker L., Akond A.S.M.G., Ali M.B., Oba S. (2010). Biomass yield and accumulations of bioactive compounds in red amaranth (*Amaranthus tricolor* L.) grown under different colored shade polyethylene in spring season. Sci. Hortic..

[17] Biddington N.L., Thomas T.H. (1973). A modified *Amaranthus* betacyanin bioassay for the rapid determination of cytokinins in plant extracts. Planta.

[18] Bianco-Colomas J. (1980). Qualitative and quantitative aspects of betalains biosynthesis in *Amaranthus caudatus* L. var. pendula seedlings. Planta.

[19] Nazmul M., Bhuiyan H., Adachi T. (2003). Stimulation of betacyanin synthesis through exogenous methyl jasmonate and other elicitors in suspension-cultured cells of *Portulaca*. J. Plant Physiol..

[20] Shin K.S., Murthy H.N., Heo J.W., Paek K.Y. (2003). Induction of betalain pigmentation in hairy roots of red beet under different radiation sources. Biol. Plant..

[21] Thomas V., Mwafaq I., Juergen S., Victor W., Manfred N., Dieter S. (1999). Light-induced betacyanin and flavonol accumulation in bladder cells of *Mesembryanthemum crystallinum*. Phytochemistry.

[22] Zhao S.Z., Sun H.Z., Chen M., Wang B.S. (2010). Light-regulated betacyanin accumulation in euhalophyte *Suaeda salsa* calli. Plant Cell Tissue Org..

[23] Jain G., Gould K.S. (2015). Functional significance of betalain biosynthesis in leaves of *Disphyma australe* under salinity stress. Environ. Exp. Bot..

[24] Jain G., Schwinn K.E., Gould K.S. (2015). Betalain induction by L-DOPA application confers photoprotection to saline-exposed leaves of *Disphyma australe*. New Phytol..

[25] Solovchenko A.E., Merzlyak M.N. (2008). Screening of visible and UV radiation as a photoprotective mechanism in plants. Russ. J. Plant Physiol..

[26] Lakhotia P., Singh K.P., Singh S.K., Singh M.C., Prasad K.V., Swaroop K. (2014). Influence of biotic and abiotic elicitors on production of betalain pigments in *bougainvillea* callus cultures. Indian J. Hortic..

[27] Han X.H., Gao Z.J., Xiao X.G. (2009). Enzymes and genes involved in the betalain biosynthesis in higher plants. Afr. J. Biotechnol..

[28] Gandia-Herrero F., Garcia-Carmona F. (2013). Biosynthesis of betalains: Yellow and violet plant pigments. Trends Plant Sci..

[29] Dieter S., Thomas V., Schliemann W. (2003). Recent advances in betalain research. Phytochemistry.

[30] Harris N.N., Javellana J., Davies K.M., Lewis D.H., Jameson P.E., Deroles S.C., Calcott K.E., Gould K.S., Schwinn K.E. (2012). Betalain production is possible in anthocyanin-producing plant species given the presence of DOPA-dioxygenase and L-DOPA. BMC Plant Biol..

[31] Nakatsuka T., Yamada E., Takahashi H., Imamura T., Suzuki M., Ozeki Y., Tsujimura I., Saito M., Sakamoto Y., Sasaki N. (2013). Genetic engineering of yellow betalain pigments beyond the species barrier. Sci. Rep..

[32] Gandia-Herrero F., Escribano J., Garcia-Carmona F. (2005). Betaxanthins as substrates for tyrosinase. An approach to the role of tyrosinase in the biosynthetic pathway of betalains. Plant Physiol..

[33] Gandia-Herrero F., Escribano J., Garcia-Carmona F. (2005). Characterization of the monophenolase activity of tyrosinase on betaxanthins: the tyramine-betaxanthin/dopamine-betaxanthin pair. Planta.

[34] Joy R.W.I., Sugiyama M., Fukuda H., Komamine A. (1995). Cloning and characterization of polyphenol oxidase cDNAs of *Phytolacca americana*. Plant Physiol..

[35] Hind G., Marshak D.R., Coughlan S.J. (1995). Spinach thylakoid polyphenol oxidase: Cloning, characterization, and relation to a putative protein kinase. Biochemistry.

[36] Ma H., Zhu H.Y., Li L.L., Chen L.J., Guo Z.F., Zhong M. (2013). Cloning and sequence analysis of polyphenol oxidase gene of *Suaeda salsa*. Guangdong Agric. Sci..

[37] Yu Z.H., Han Y.N., Xiao X.G. (2015). A PPO promoter from betalain-producing red swiss chard, directs petiole- and root-preferential expression of foreign gene in anthocyanins-producing plants. Int. J. Mol. Sci..

[38] Casique-Arroyo G., Martinez-Gallardo N., de la Vara L.G., Delano-Frier J.P. (2014). Betacyanin biosynthetic genes and enzymes are differentially induced by (a)biotic stress in *Amaranthus hypochondriacus*. PLoS ONE.

[39] Christinet L., Burdet F., Zaiko M., Hinz U., Zryd J.P. (2004). Characterization and functional identification of a novel plant 4,5-extradiol dioxygenase involved in betalain pigment biosynthesis in *Portulaca grandiflora*. Plant Physiol..

[40] Takahashi K., Takamura E., Sakuta M. (2009). Isolation and expression analysis of two DOPA dioxygenases in *Phytolacca americana*. Z. Naturforsch. C.

[41] Zhao S.Z., Sun H.Z., Gao Y., Sui N., Wang B.S. (2011). Growth regulator-induced betacyanin accumulation and dopa-4,5-dioxygenase (*DODA*) gene expression in euhalophyte *Suaeda salsa* calli. In Vitro Cell. Dev. Biol.-Plant.

[42] Yuan R. (2008). The Cloning and Functional Analysis of 4,5-DOPA-Dioxygenase in *Suaeda salsa*. Master’s Thesis.

[43] Gandia-Herrero F., Garcia-Carmona F. (2012). Characterization of recombinant *β vulgaris* 4,5-DOPA-extradiol-dioxygenase active in the biosynthesis of betalains. Planta.

[44] Stintzing F.C., Herbach K.M., Mosshammer M.R., Carle R., Yi W.G., Sellappan S., Akoh C.C., Bunch R., Felker P. (2005). Color, betalain pattern, and antioxidant properties of cactus pear (*Opuntia spp.*) clones. J. Agric. Food Chem..

[45] Chung H., Schwinn K.E., Ngo H.M., Lewis D.H., Massey B., Calcottt K.E., Crowhurst R., Joyce D.C., Gould K.S., Davies K.M. (2015). Characterisation of betalain biosynthesis in *Parakeelya* flowers identifies the key biosynthetic gene DOD as belonging to an expanded *LigB* gene family that is conserved in betalain-producing species. Front. Plant Sci..

[46] Sasaki N., Abe Y., Goda Y., Adachi T., Kasahara K., Ozeki Y. (2009). Detection of DOPA 4,5-dioxygenase (DOD) activity using recombinant protein prepared from *Escherichia coli* cells harboring cDNA encoding *DOD* from *Mirabilis jalapa*. Plant Cell Physiol..

[47] Hatlestad G.J., Sunnadeniya R.M., Akhavan N.A., Gonzalez A., Goldman I.L., McGrath J.M., Lloyd A.M. (2012). The beet R locus encodes a new cytochrome P450 required for red betalain production. Nat. Genet..

[48] Suzuki M., Miyahara T., Tokumoto H., Hakamatsuka T., Goda Y., Ozeki Y., Sasaki N. (2014). Transposon-mediated mutation of *CYP76AD3* affects betalain synthesis and produces variegated flowers in four o’clock (*Mirabilis jalapa*). J. Plant Physiol..

[49] Yang Y., Moore M.J., Brockington S.F., Soltis D.E., Wong G.K.S., Carpenter E.J., Zhang Y., Chen L., Yan Z., Xie Y. (2015). Dissecting molecular evolution in the highly diverse plant clade Caryophyllales using transcriptome sequencing. Mol. Biol. Evol..

[50] Brockington S.F., Yang Y., Gandia-Herrero F., Covshoff S., Hibberd J.M., Sage R.F., Wong G.K.S., Moore M.J., Smith S.A. (2015). Lineage-specific gene radiations underlie the evolution of novel betalain pigmentation in Caryophyllales. New Phytol..

[51] Isayenkova J., Wray V., Manfred N., Strack D., Thomas V. (2006). Cloning and functional characterisation of two regioselective flavonoid glucosyltransferases from *Beta vulgaris*. Phytochemistry.

[52] Noguchi A., Kunikane S., Homma H., Liu W., Sekiya T., Hosoya M., Kwon S., Ohiwa S., Katsuragi H., Nishino T. (2009). Identification of an inducible glucosyltransferase from *Phytolacca americana* L. cells that are capable of glucosylating capsaicin. Plant Biotechnol. Nar..

[53] Thomas V., Rudi G., Dieter S. (1999). Cloning and expression of a cDNA encoding betanidin 5-*O*-glucosyltransferase, a betanidin- and flavonoid-specific enzyme with high homology to inducible glucosyltransferases from the Solanaceae. Plant J..

[54] Thomas V. (2002). Substrate specificity and sequence analysis define a polyphyletic origin of betanidin 5- and 6-*O*-glucosyltransferase from *Dorotheanthus bellidiformis*. Planta.

[55] Sasaki N., Wada K., Koda T., Kasahara K., Adachi T., Ozeki Y. (2005). Isolation and characterization of cDNAs encoding an enzyme with glucosyltransferase activity for cyclo-DOPA from four o’clocks and feather cockscombs. Plant Cell Physiol..

[56] Hatlestad G.J., Akhavan N.A., Sunnadeniya R.M., Elam L., Cargile S., Hembd A., Gonzalez A., McGrath J.M., Lloyd A.M. (2015). The beet Y locus encodes an anthocyanin MYB-like protein that activates the betalain red pigment pathway. Nat. Genet..

[57] Des Marais D.L. (2015). To betalains and back again: A tale of two pigments. New Phytol..

[58] Lim T.K. (2012). *Hylocereus* *polyrhizus*. Edible Medicinal and Non-Medicinal Plants.

[59] Lim T.K. (2012). *Hylocereus* *undatus*. Edible Medicinal and Non-Medicinal Plants.

[60] Adnan L., Osman A., Hamid A.A. (2011). Antioxidant activity of different extracts of red pitaya (*Hylocereus polyrhizus*) seed. Int. J. Food Prop..

[61] Zhuang Y.L., Zhang Y.F., Sun L.P. (2012). Characteristics of fibre-rich powder and antioxidant activity of pitaya (*Hylocereus undatus*) peels. Int. J. Food Sci. Technol..

[62] Garcia-Cruz L., Valle-Guadarrama S., Salinas-Moreno Y., Joaquin-Cruz E. (2013). Physical, chemical, and antioxidant activity characterization of pitaya (*Stenocereus pruinosus*) fruits. Plant Food Hum. Nutr..

[63] Wu L.C., Hsu H.W., Chen Y.C., Chiu C.C., Lin Y.I., Ho J.A. (2006). Antioxidant and antiproliferative activities of red pitaya. Food Chem..

[64] Tenore G.C., Novellino E., Basile A. (2012). Nutraceutical potential and antioxidant benefits of red pitaya (*Hylocereus polyrhizus*) extracts. J. Funct. Foods.

[65] Wybraniec S., Stalica P., Jerz G., Klose B., Gebers N., Winterhalter P., Sporna A., Szaleniec M., Mizrahi Y. (2009). Separation of polar betalain pigments from cacti fruits of *Hylocereus polyrhizus* by ion-pair high-speed countercurrent chromatography. J. Chromatogr. A.

[66] Lim S.D., Yusof Y.A., Chin N.L., Talib R.A., Endan J., Aziz M.G. (2011). Effect of extraction parameters on the yield of betacyanins from pitaya fruit (*Hylocereus polyrhizus*) pulps. J. Food Agric. Environ..

[67] Suh D.H., Lee S., Heo D.Y., Kim Y., Cho S.K., Lee S., Lee C.H. (2014). Metabolite profiling of red and white pitayas (*Hylocereus polyrhizus* and *Hylocereus undatus*) for comparing betalain biosynthesis and antioxidant activity. J. Agric. Food Chem..

[68] Hua Q.Z., Chen C.J., Chen Z., Chen P.K., Ma Y.W., Wu J.Y., Zheng J., Hu G.B., Zhao J.T., Qin Y.H. (2016). Transcriptomic analysis reveals key genes related to betalain biosynthesis in pulp coloration of *Hylocereus polyrhizus*. Front. Plant Sci..

[69] Casado-Vela J., Jose Martinez-Esteso M., Rodriguez E., Borras E., Elortza F., Bru-Martinez R. (2010). iTRAQ-based quantitative analysis of protein mixtures with large fold change and dynamic range. Proteomics.

[70] Tian Q., Stepaniants S.B., Mao M., Weng L., Feetham M.C., Doyle M.J., Yi E.C., Dai H.Y., Thorsson V., Eng J. (2004). Integrated genomic and proteomic analyses of gene expression in mammalian cells. Mol. Cell Proteom..

[71] Rossouw D., van den Dool A.H., Jacobson D., Bauer F.F. (2010). Comparative transcriptomic and proteomic profiling of industrial wine yeast strains. Appl. Environ. Microb..

[72] Carp M.-J., Gepstein S., Gan S. (2007). Genomics and proteomics of leaf senescence. Senescence Processes in Plants.

[73] Jamaludin N.A., Ding P., Hamid A.A. (2011). Physico-chemical and structural changes of red-fleshed dragon fruit (*Hylocereus polyrhizus*) during fruit development. J. Sci. Food Agric..

[74] Shimada S., Inoue Y.T., Sakuta M. (2005). Anthocyanidin synthase in non-anthocyanin-producing caryophyllales species. Plant J..

