# Supplementary material for: Proteomic Analysis of Hylocereus polyrhizus Reveals Metabolic Pathway Changes
Source: Int J Mol Sci. 2016 Sep 28;17(10):1606. doi: 10.3390/ijms17101606 (PMC5085639; doi:10.3390/ijms17101606)
Supplement: Supplementary file 1 [file ijms-17-01606-s001.zip › ijms-141528-Supplementary Materials/ijms-141528-supplementary.pdf]

Supplementary Materials: Proteomic Analysis of *Hylocereus polyrhizus* Reveals Metabolic Pathway Changes

Qingzhu Hua, Qianjun Zhou, Susheng Gan, Jingyu Wu, Canbin Chen, Jiaqiang Li, Yaoxiong Ye, Jietang Zhao, Guibing Hu and Yonghua Qin

Table S1. Differentially expressed on genes but not on proteins (*p*-value < 0.05, fold change (pro) < 1.3, fold change (RNA) > 2).

Gene Ontology (GO) Classification	Number (No.) Protein
**Biological Process (BP)**	
GO:0055114//oxidation-reduction process	18
GO:0006184//GTP catabolic process	4
GO:0015937//coenzyme A biosynthetic process	2
GO:0006526//arginine biosynthetic process	2
GO:0006032//chitin catabolic process	1
GO:0016998//cell wall macromolecule catabolic process	1
GO:0006839//mitochondrial transport	1
GO:0009156//ribonucleoside monophosphate biosynthetic process	1
**Molecular Function (MF)**	
GO:0016705//oxidoreductase activity, acting on paired donors, with incorporation or reduction of molecular oxygen	9
GO:0005506//iron ion binding	9
GO:0020037//heme binding	9
GO:0045735//nutrient reservoir activity	3
GO:0004055//argininosuccinate synthase activity	2
GO:0004594//pantothenate kinase activity	2
GO:0008184//glycogen phosphorylase activity	2
GO:0016811//hydrolase activity, acting on carbon-nitrogen (but not peptide) bonds, in linear amides	2
GO:0043531//ADP binding	2
GO:0003868//4-hydroxyphenylpyruvate dioxygenase activity	1
GO:0004314//[acyl-carrier-protein] *S*-malonyltransferase activity	1
GO:0004556//alpha-amylase activity	1
GO:0004568//chitinase activity	1
GO:0008061//chitin binding	1
GO:0030674//protein binding, bridging	1
GO:0051015//actin filament binding	1
GO:0003840//gamma-glutamyltransferase activity	1
GO:0008113//peptide-methionine (*S*)-*S*-oxide reductase activity	1
GO:0016701//oxidoreductase activity, acting on single donors with incorporation of molecular oxygen	1
GO:0016790//thiolester hydrolase activity	1
**Cellular Component (CC)**	
GO:0043234//protein complex	2
GO:0005874//microtubule	2
GO:0009538//photosystem I reaction center	1
GO:0031966//mitochondrial membrane	1
GO:0009522//photosystem I	1
GO:0042555//MCM complex	1

Table S2. Differentially expressed on genes but on proteins (*p*-value < 0.05, fold change (pro) < 1.3, fold change (RNA) > 2).

KEGG Pathway	No. Protein
ko00330 Arginine metabolism	5
ko00250 Alanine aspartate glutamate metabolism	4
ko00620 Pyruvate metabolism	4
ko00710 Carbon fixation in photosynthetic organisms	4
ko00061 Fatty acid biosynthesis	3
ko00910 Nitrogen metabolism	3
ko01004 Lipid biosynthesis proteins	3
ko00199 Cytochrome P450	2
ko00130 Ubiquinone terpenoid-quinone biosynthesis	2
ko00770 Pantothenate biosynthesis	2
ko04540 Gap junction	2
ko00905 Brassinosteroid biosynthesis	1

Table S3. Negative correlation (*p*-value < 0.05, FC (pro) > 1.5, FC (RNA) > 2).

GO Classification	No. Protein
**Biological Process (BP)**	
GO:0042549//photosystem stabilization	1
GO:0006090//pyruvate process	1
GO:0006231//dTMP biosynthetic process	1
GO:0006545//glycine process	1
GO:0006869//lipid transport	1
GO:0009165//nucleotide biosynthetic process	1
**Molecular Function (MF)**	
GO:0003677//DNA binding	4
GO:0008234//cysteine-type peptidase activity	2
GO:0009055//electron carrier activity	2
GO:0003756//protein disulfide activity	1
GO:0005337//nucleoside transmembrane transporter activity	1
GO:0008146//sulfotransferase activity	1
GO:0050242//pyruvate, phosphate dikinase activity	1
GO:0004146//dihydrofolate activity	1
GO:0004799//thymidylate activity	1
GO:0004866//endopeptidase inhibitor activity	1
GO:0004869//cysteine-type endopeptidase inhibitor activity	1
GO:0016671//oxidoreductase activity	1
**Cellular Component (CC)**	
GO:0009279//cell outer membrane	1
GO:0009654//oxygen evolving complex	1
GO:0019898//extrinsic to membrane	1
GO:0009523//photosystem II	1

Table S4. Negative correlation (*p*-value < 0.05, FC (pro) > 1.5, FC (RNA) > 2).

KEGG Pathway	No. Protein
ko04142 Lysosome	4
ko04145 Phagosome	2
ko04210 Apoptosis	1
ko04115 p53 signaling pathway	1
ko04712 Circadian plant	1

Table S5. Gradient used for grading of polypeptides.

**Time (min)**	**0**	**2**	**17**	**29**	**30**	**31**	**36**	**37**	**57**
Buffer A (%)	96	92	80	65	55	5	5	96	96
Buffer B (%)	4	8	20	35	45	95	95	4	4

Table S6. Gradient used for LC-MS/MS.

**Time (min)**	**0**	**5**	**6**	**48**	**62**	**65**	**70**	**70.5**	**90**
Buffer A (%)	97	97	94	83	70	20	20	97	97
Buffer B (%)	3	3	6	17	30	80	80	3	3

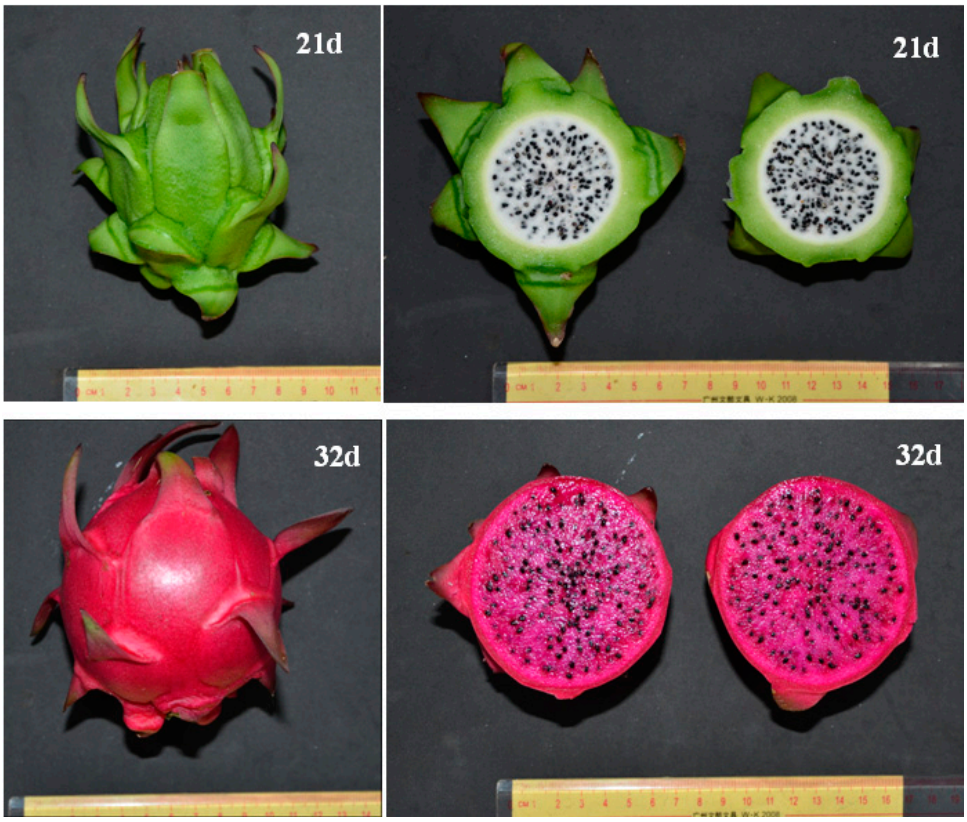

Figure S1. Pictures of pitaya fruits (entire and cut in to observe internal color) collected on the 21st day (white pulp) and 32rd day (red white).
